# Supplementary material for: Order and information in the patterns of spinning magnetic micro-disks at the air-water interface
Source: Sci Adv. 2022 Jan 14;8(2):eabk0685. doi: 10.1126/sciadv.abk0685 (PMC8759740; doi:10.1126/sciadv.abk0685)
Supplement: Supplementary file 1 — Figs. S1 to S10 Tables S1 and S2 Legends for movies S1 to S6 [file sciadv.abk0685_sm.pdf]

Science Advances
AAAS

Supplementary Materials for

Order and information in the patterns of spinning magnetic micro-disks at the air-water interface

Wendong Wang*, Gaurav Gardi, Paolo Malgaretti, Vimal Kishore, Lyndon Koens, Donghoon Son, Hunter Gilbert, Zongyuan Wu, Palak Harwani, Eric Lauga, Christian Holm, Metin Sitti*

*Corresponding author. Email: wendong.wang@sjtu.edu.cn (W.W.); sitti@is.mpg.de (M.S.)

Published 14 January 2022, *Sci. Adv.* **8**, eabk0685 (2022)
DOI: 10.1126/sciadv.abk0685

The PDF file includes:

Figs. S1 to S10
Tables S1 and S2
Legends for movies S1 to S6

Other Supplementary Material for this manuscript includes the following:

Movies S1 to S6

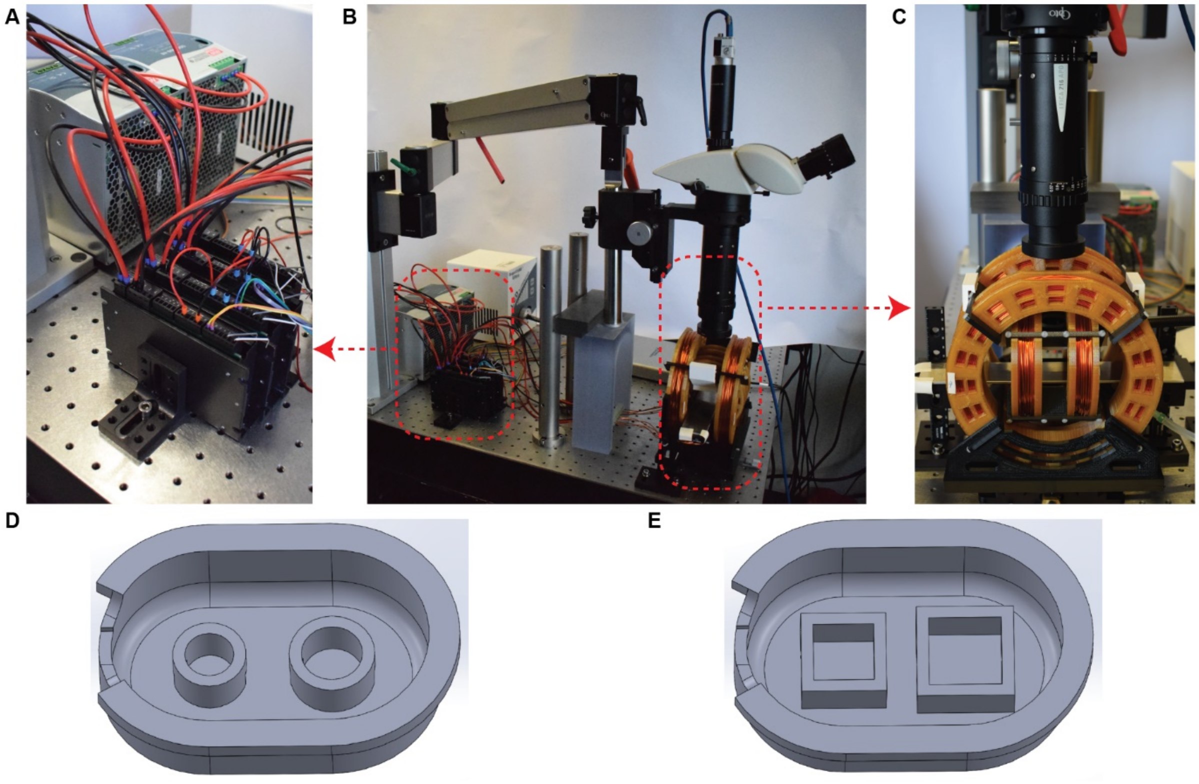

Fig. S1. Experimental setup.

(**A**) The power supplies (Mean Well, SDR – 960 – 48) and motor servo controllers used as current controllers (Maxon ESCON 70/10). (**B**) Overview of the custom-made two-axis Helmholtz coil system and the imaging setup. The system consists of magnetic coils, an imaging system (Leica Z16 APO + SugarCUBE Ultra illuminator), current amplifiers, and analogue signal generators (National Instruments USB-6363). (**C**) Custom-designed magnetic coils (coils of 10 cm and 16 cm in diameter) and the imaging system. (**D** – **E**) Containers and arenas used in the experiments. Arenas refer to the space surrounded by circles or features of other shapes inside the oval-shaped container. The size of the container is 52 x 32 x 10 mm, with 8 mm radius filets around the four corners. (**D**) The arena for experiments on pairwise interaction. The diameters of the left and the right rings are 8 mm and 10 mm, respectively. (**E**) The arenas for many-disks and tiling experiments. The inside edges for the left and the right squares are 10 mm and 15 mm, respectively.

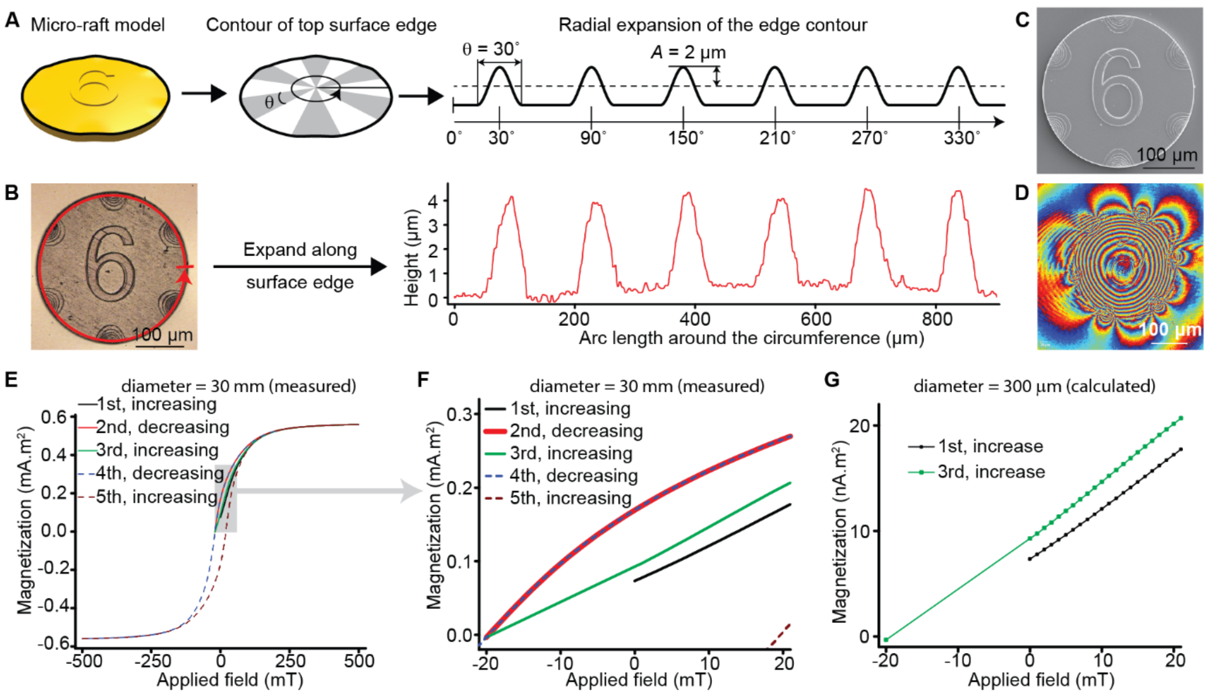

Fig. S2. The design and characterization of a single micro-disk.

(**A**) The parametric model of one micro-disk. Its surface edge has six cosinusoidal profiles. Each profile has an arc angle θ of 30° and an amplitude A of 2 µm. (**B**) The laser confocal image overlapped with an optical image and the expanded edge height profile showing the six cosinusoidal profiles around the edge. (**C**) Scanning electron microscope image of one representative micro-disk. (**D**) Digital holographic microscope phase image of one micro-disk on the air-water interface. The deformation of the interface shows 6-fold symmetry. (**E**) Magnetization of 500 nm cobalt thin film sputtered on 30 mm coverslip. (**F**) Zoomed-in view of the grey region in (e). (**G**) The magnetization of 500 nm cobalt thin film on micro-disks of 300 µm in diameter. It is calculated from (f). For the measurement of the magnetization, B-field strength changed as the following: (1) 0 to 500 mT, (2) 500 mT to -20 mT, (3) -20 mT to 500 mT, (4) 500 mT to -500 mT, and (5) -500 mT to 500 mT.

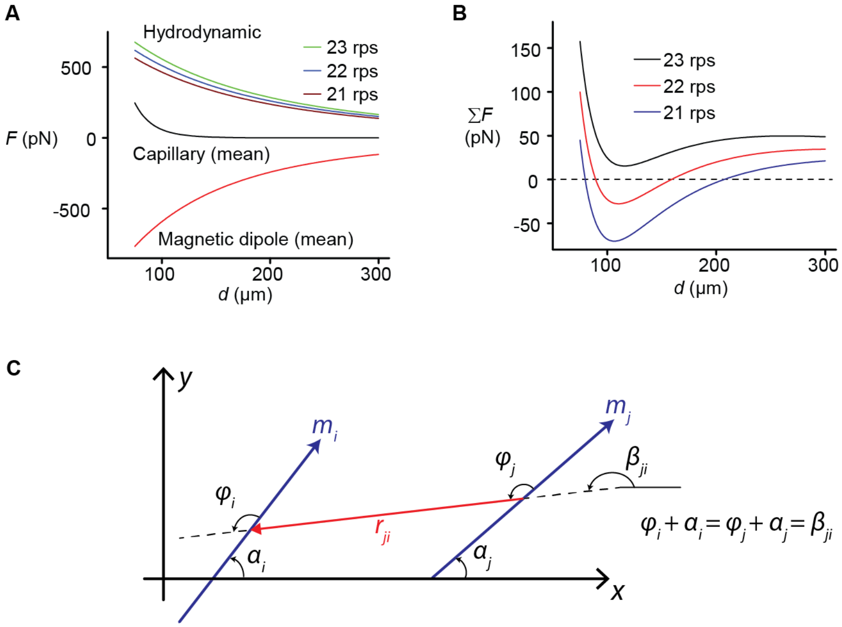

Fig. S3 Increasing hydrodynamic lift force is responsible for the decoupling transition and scheme for the magnetic dipole-dipole interaction.

(**A**) Three main forces as a function of the edge-edge distance *d*, calculated from the numerical model. Positive and negative values correspond to repulsion and attraction, respectively. Because capillary force and magnetic dipole-dipole force are angle-dependent, their angle-averaged values are used in the plot. (**B**) Sum of the three main forces in the range of 21 – 23 rps. For 21 rps and 22 rps, each sum of all forces has a stable stationary point (the point where the curve crosses the *x*-axis with a negative slope), and this stationary point corresponds to the steady-state observed in the experiment. At 23 rps, the sum of all forces becomes positive and hence repulsive at all separation distances, so the two micro-disks decouple and move away from each other. (**C**) Scheme of the magnetic dipole-dipole interaction. The blue arrows are the directions of the magnetic dipole of micro-disks. The red arrow is the centre-to-centre axis of micro-disks.

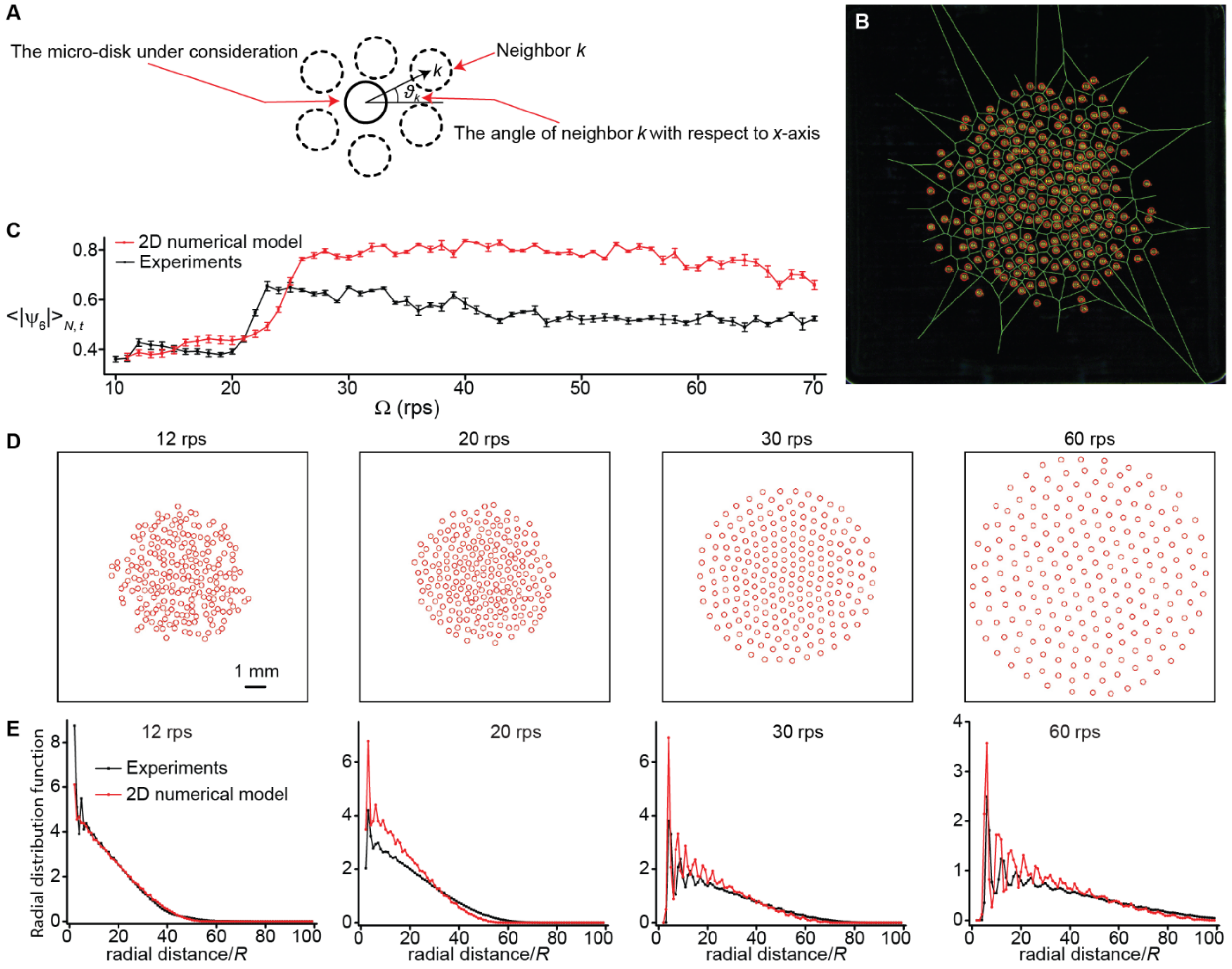

Fig. S4 Hexatic order parameters for the patterns produced by 2D numerical model of many micro-disks.

(**A**) Schematics showing the calculation of hexatic order parameter $\psi_6 = \sum_k \exp\,(i6\vartheta_k)\,/K$. K is the number of one micro-disk's neighbours; k is the neighbour index; ϑ_k is the polar angle of the vector from the micro-disk to its neighbour k. Neighbours are defined by Voronoi tessellation. (**B**) An example of the Voronoi tessellation. (**C**) The averaged norms of the hexatic order parameters $<|\psi_6|>_{N,t}$ as a function of the rotation speed of the magnetic field **Ω**. The 2D numerical model is based on the equations of motion of many micro-disks. The experimental data are the same as in Fig. 1E and are added for comparison. The model captures the main disorder-order transition at ~23 – 25 rps. (**D**) Representative simulated patterns at 12, 20, 30, and 60 rps based on the 2D numerical model. (**E**) The radial distribution function of the experimental patterns and the patterns obtained from the 2D numerical model. The experimental data were averaged over 75 frames (1 s), and the simulation data were averaged over 100 frames.

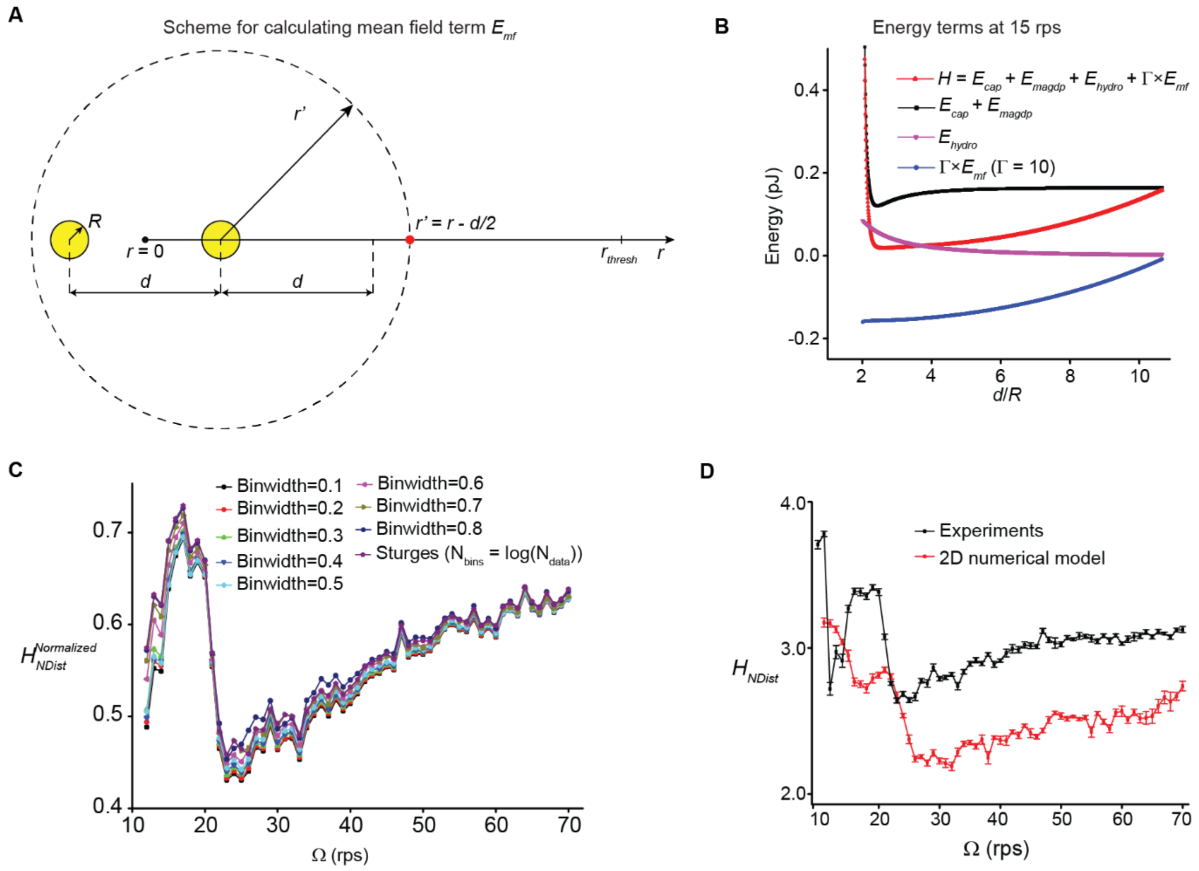

Fig. S5 Scheme for calculating the mean-field term E_{mf}, Energy terms at 15 rps, the effect of bin widths on the entropy by neighbour distances, and comparison of the entropies by neighbour distances calculated from experimental data and from 2D numerical model.

(A) The geometry used for the construction of a mean-field energy term to account for the interactions between one pair of micro-disks with all the rest micro-disks. When considering the micro-disk on the right, the area density of micro-disks is assumed to be uniform from $r = 3d/2$ to $r = r_{thresh}$. r_{thresh} is half of the edge length of the physical boundary. (B) The energy terms at 15 rps. Hamiltonian-like energy function and its components, including capillary energy (angle-averaged), magnetic dipole-dipole energy (angle-averaged), hydrodynamic energy, and effective boundary energy. (C) The normalized adjusted Shannon entropy vs spin speeds. The adjusted H_{Dist} was calculated according to $H_{NDist} = -\sum_i p_i \log(p_i/w_i)$, where w_i is the bin width. This adjusted Shannon entropy was then normalized by the logarithm of the sum of bin widths: $H_{NDist}^{Normalized} = \frac{-\sum_i p_i \log\left(\frac{p_i}{w_i}\right)}{\log(\sum_i w_i)}$. The base of the logarithm is 2. The fact that all the curves collapse into one curve suggests that within the range of bin width (0.1 – 0.8R), the bin widths do not affect the result. (**D**) The entropies by neighbour distances H_{NDist} as a function of the rotation speed of the magnetic field **Ω**. Comparison between the experimental values and the values from the 2D numerical model shows that the 2D numerical model captures the main transition at ~23 rps and the additional transition at ~15 rps. However, it does not capture the dip at around ~13 rps. The patterns based on the 2D numerical model have lower H_{NDist} than the experimental values, probably because they are not affected by the presence of environmental noise in experiments.

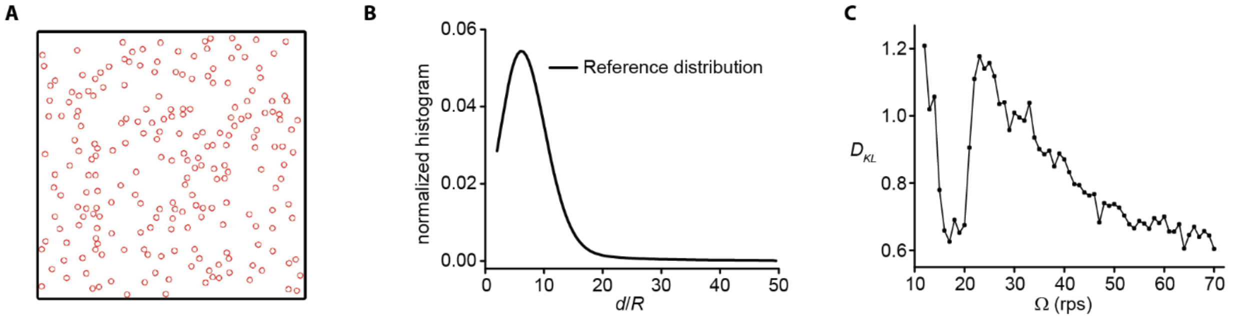

Fig. S6 Reference distribution of randomly positioned micro-disks and KL divergence as a measure of extra information embedded in neighbour distance distributions.

(**A**) A representative pattern of randomly positioned micro-disks. The micro-disks are subject to two constraints: (1) they do not non-overlap, and (2) they are within the boundary of the arena. (**B**) The averaged distribution of neighbour distances of 100, 000 randomly-generated patterns. (**C**) The Kullback-Leibler divergence $D_{KL}(P \parallel Q)$ between the distributions of experimental neighbour distances P(x) and the reference distribution Q(x). $D_{KL}(P \parallel Q) = \Sigma P(x) \ln(P(x)/Q(x))$.

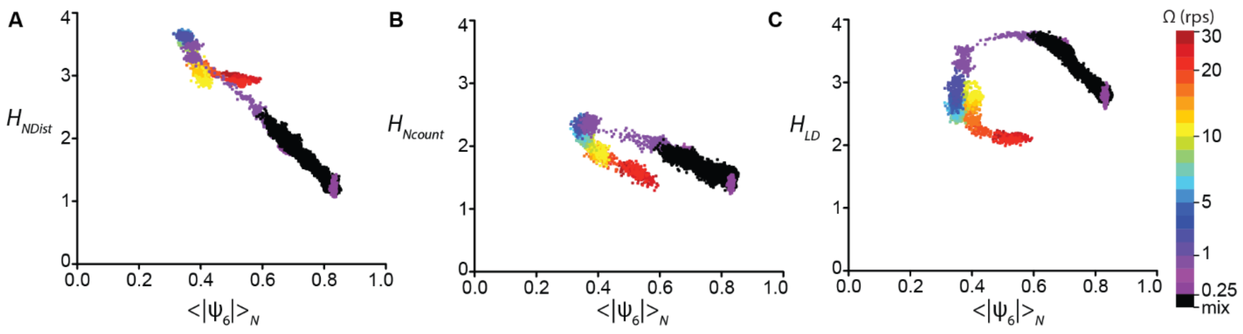

Fig. S7. Pearson correlation of different entropies with the hexatic order parameter.

(**A**) The Shannon entropy by neighbour distances H_{NDist} vs the number average of the norms of the hexatic order parameter $<|\psi_6|>_N$. The two quantities are almost entirely anti-correlated. The Pearson correlation coefficient is -0.99. (**B**) The Shannon entropy by neighbour counts vs the number average of the norms of the hexatic order parameter $<|\psi_6|>_N$. The Pearson correlation coefficient is -0.84. **(C)** The Shannon entropy by local densities vs the number average of the norms of the hexatic order parameter $<|\psi_6|>_N$. The Pearson correlation coefficient is 0.15.

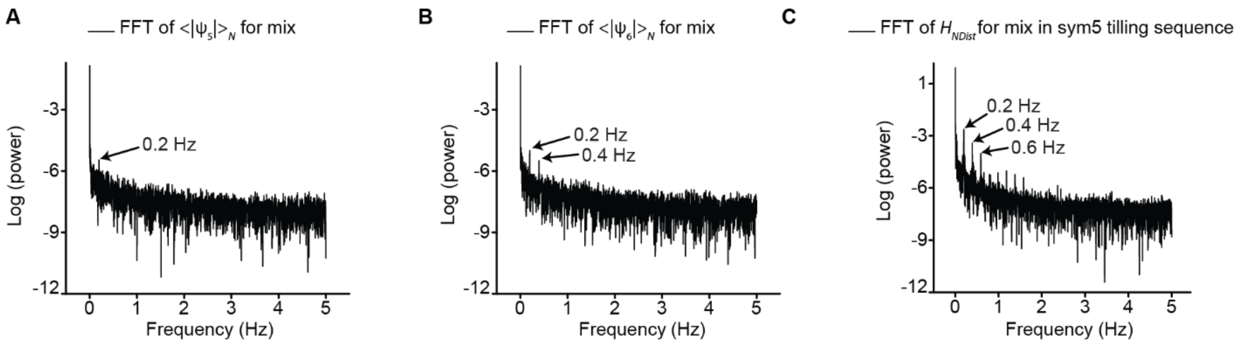

Fig. S8. Fourier spectra of $<|\psi_5|>_N$, $<|\psi_6|>_N$, and H_{NDist} for the mixing period in the tiling of micro-disks with 5-fold symmetry.

(**A**) The Fourier spectrum of $<|\psi_5|>_N$ shows a small peak at 0.2 Hz, corresponding to the 5s period of Mix sequences used in the experiments. (**B**) the Fourier spectrum of $<|\psi_6|>_N$, showing stronger peaks at 0.2 Hz, and a second harmonics at 0.4 Hz. (**C**) The Fourier spectrum of H_{NDist}, showing strong peaks at 0.2 Hz (fundamental frequency), 0.4 Hz (second harmonic), 0.6 Hz (third harmonic) and several small peaks at higher order harmonics. The time series of these spectra are shown in Fig. 4F.

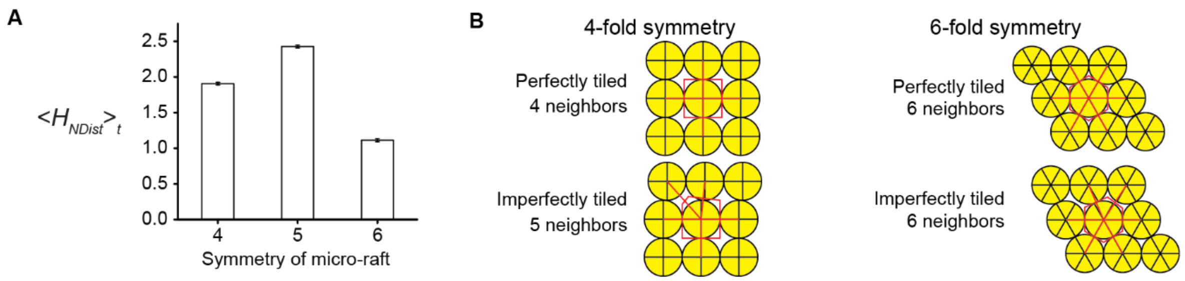

Fig. S9. Comparison of the degree of orders in crystal-like patterns of different symmetries. (**A**) Comparison of the smallest entropies by neighbour distances for three different symmetries of micro-disks. (**B**) The schematics show that a slight misalignment in 4-fold symmetry tiling gives an additional neighbour (and an additional neighbour distance), whereas a slight misalignment in 6-fold symmetry tiling preserves the number of neighbours. It explains why the best crystal-like pattern for 6-fold symmetry has the lowest entropy.

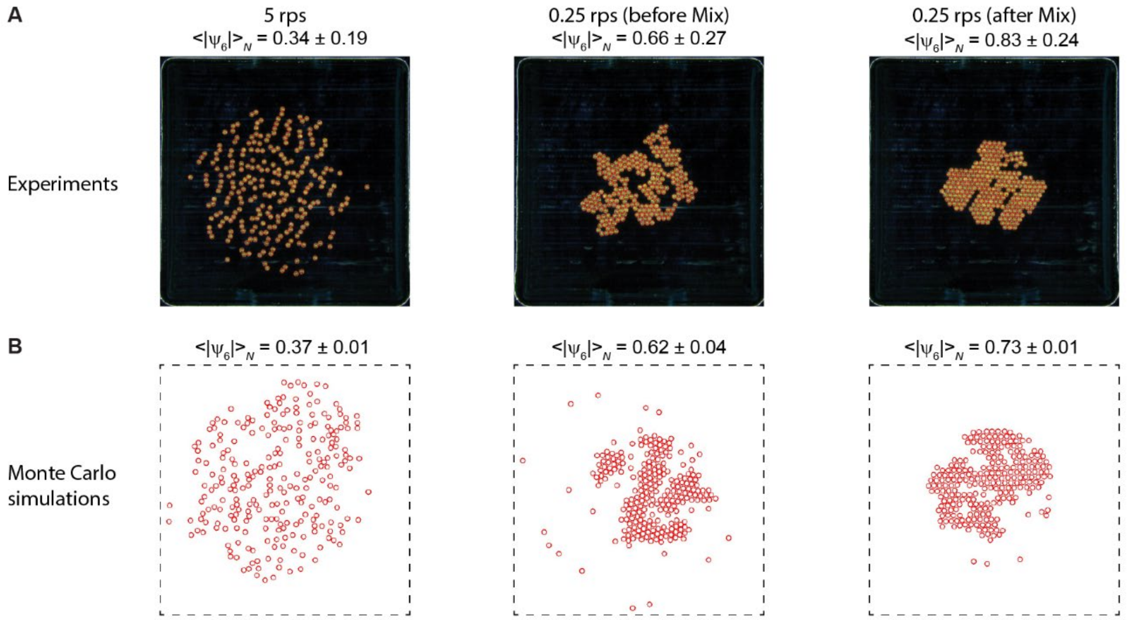

Fig. S10. Monte Carlo simulation of patterns from a single frame at low spin speeds.

(**A**) Experimental patterns at 5 rps, 0.25 rps (before Mix), and 0.25 rps (after Mix). (**B**) The corresponding patterns from the Monte Carlo simulations.

Table S1: Summary of Monte Carlo simulations results on steady-state patterns

	Simulations				*Experiments*			
	12 rps	*20 rps*	*30 rps*	*60 rps*	*12 rps*	*20 rps*	*30 rps*	*60 rps*
Max $<\|\psi_6\|>_N$	0.53	0.45	0.62	0.56	0.46	0.42	0.67	0.56
Average $<\|\psi_6\|>_N$	0.47± 0.02	0.39 ± 0.01	0.55 ± 0.02	0.52 ± 0.02	0.43 ± 0.01	0.38 ± 0.01	0.61 ± 0.02	0.52 ± 0.02
Average $\|<\psi_6>_N\|$	0.07 ± 0.04	0.03 ± 0.02	0.14 ± 0.07	0.09 ± 0.05	0.05	0.04	0.19	0.08
min KLDiv NDist	0.02 ± 0.003	0.02 ± 0.003	0.02 ± 0.005	0.027 ± 3e-4	-	-	-	-
ref KLDiv NDist	0.05	0.05	0.025	0.05	-	-	-	-
min KLDiv X	0.5 ± 0.2	0.3 ± 0.2	0.4 ± 0.3	0.32 ± 0.08	-	-	-	-
ref KLDiv X	0.1	0.1	0.05	0.1	-	-	-	-
min KLDiv Y	0.7 ± 0.2	0.2 ± 0.06	0.4 ± 0.4	0.3 ± 0.09	-	-	-	-
ref KLDiv Y	0.1	0.1	0.05	0.1	-	-	-	-
min KLDiv Odist	0.98 ± 0.05	0.4 ± 0.2	0.7 ± 0.3	0.34 ± 0.001	-	-	-	-
ref KLDiv Odist	0.25	0.25	0.25	0.25	-	-	-	-
# accepted based on Prob	0.0 ± 0.0	0.0 ± 0.0	0.0 ± 0.0	0.0 ± 0.0	-	-	-	-
# runs	7	7	7	7	-	-	-	-

Table S2: Summary of Monte Carlo simulations results on single-frame patterns at low spin speeds

	Simulations			***Experiments***		
	5 rps	*0.25 rps (before Mix)*	*0.25 rps (after Mix)*	*5 rps*	*0.25 rps (before Mix)*	*0.25 rps (after Mix)*
Max $<\lvert\psi_6\rvert>_N$	0.49	0.67	0.77	0.34	0.66	0.83
Average $<\lvert\psi_6\rvert>_N$	0.37± 0.01	0.62± 0.04	0.73 ± 0.01	0.34	0.66	0.83
Average $\lvert<\psi_6>_N\rvert$	0.030 ± 0.01	0.16± 0.06	0.49 ± 0.16	0.035	0.10	0.81
min KLDiv NDist	0.0112 ± 0.0001	0.04 ± 0.02	0.014 ± 0.004	-	-	-
ref KLDiv NDist	-	-	-	-	-	-
# accepted based on Prob	0.0 ± 0.0	0.0 ± 0.0	0.0 ± 0.0	-	-	-
# runs	5	7	7	-	-	-

Description of supplementary movies

Movie S1. Pairwise interactions.

This video contains representative experimental and simulation videos of pairwise interactions, showing assembled, orbiting, and decoupled states. In the processed videos, the blue lines correspond to the positive *x*-directions of micro-disks in the first video frame. In the simulation videos, the blue lines correspond to dipole directions, and the green lines indicate the capillary peaks.

Movie S2. Representative experimental patterns – short videos

This video contains experimental patterns of 218 micro-disks at 12, 20, 30, 60 rps. Each recording is of two seconds.

Movie S3. Representative experimental patterns – long videos

This video contains experimental patterns of 218 micro-disks at 15, 20, 30, 60 rps. Each recording is of twenty seconds

Movie S4. Representative simulated patterns

This video shows simulated patterns of 218 micro-disks at 12, 20, 30, 60 rps.

Movie S5. Tiling of micro-disks of 6-fold symmetry

This video shows the tiling experiment of micro-disks with local 6-fold symmetry.

Movie S6. Tiling of micro-disks of 4-fold and 5-fold symmetry

The left part of this video shows the tiling of micro-disks with local 4-fold symmetry. The right part of this video shows the pattern transition of micro-disks with local 5-fold symmetry.
